# Quantitative proteomic analysis reveals sophisticated metabolic alteration and identifies FMNL1 as a prognostic marker in clear cell renal cell carcinoma

**DOI:** 10.7150/jca.62309

**Published:** 2021-09-09

**Authors:** Gui Ma, Zirui Wang, Junyao Liu, Shengjun Fu, Lili Zhang, Duo Zheng, Panfeng Shang, Zhongjin Yue

**Affiliations:** 1 The Second Clinical College, Lanzhou University, Lanzhou 730030, Gansu, China.; 2 Department of Urology, Lanzhou University Second Hospital, Lanzhou 730030, Gansu, China.; 3 Key Laboratory of Urological Diseases in Gansu Province, Lanzhou University Second Hospital, Lanzhou 730030, Gansu, China.

**Keywords:** ccRCC, proteomics, prognosis, FMNL1

## Abstract

**Purpose:** In this study, we have undertaken the whole proteomic analysis and got a better understanding of biological processes involved in the development and progression of ccRCC. We hope promising biomarkers can be uncovered to facilitate early diagnosis, predict the prognosis and progression, more importantly, to be applied as potential therapeutic targets.

**Experimental design:** Fresh frozen tissue samples were surgically resected from patients with local or locally advanced ccRCC. Trypsin digested proteins were analyzed using TMT-based LC-MS/MS proteomic approach, followed by bioinformatic analysis. A potential prognostic marker FMNL1 was chosen to be validated in TCGA_KIRC datasets (n=525 and 72), further validation sets (n=10 and 10) and expanded validation sets (n=81 and 16). The effects of FMNL1 on proliferation, migration and invasion were determined by colony formation, wound healing, and transwell assays in 786-O and Caki-1 cells *in vitro* study.

**Results**: A total of 657 differentially expressed proteins were identified and quantified between ccRCC and adjacent normal tissues (p-value<0.05, FC>2 or<1/2), of which 186 proteins were up-regulated and 471 proteins were down-regulated. Bioinformatic analysis showed enriched metabolic biological processes and pathways. Univariate and multivariate analysis defined FMNL1 as an independent negative prognostic marker in the TCGA datasets. High expression of FMNL1 correlated significantly with tumor stage and distant metastasis (P<0.05) both in the TCGA-KIRC datasets and expanded validation sets. Kaplan-Meier survival curve illustrated that the patients with high FMNL1 protein level had shorter OS time in the expanded validation sets (p=0.0273). *In vitro* experiments presented the functional effects of FMNL1 knockdown on the inhibition of proliferation, migration and invasion in cancer cell lines.

**Conclusion and clinical relevance:** The proteomic results uncovered sophisticated metabolic reprogramming of ccRCC and indicated that the upregulation of rate-limiting enzymes in glycolysis and mitochondrial impairment may be the cause of metabolic reprogramming in ccRCC. Moreover, FMNL1 has been identified as a promising prognostic marker, and knockdown of FMNL1 could inhibit ccRCC cell proliferation, migration and invasion, which might be used as a new effective therapeutic strategy to inhibit the progression of ccRCC.

## Introduction

Renal cell carcinoma (RCC) is one of the most common cancers in urological neoplasm that arise from renal proximal tubular epithelial cells. The global incidence of kidney cancer ranks ninth in males and fourteenth in females, and there was estimated to be approximately 403,262 new cancer cases and 175,098 cancer deaths in 2018 1. Clear cell carcinoma (ccRCC) is the most predominant histological subtype of RCC, accounting for approximately 70%-80% of renal neoplasm. Clinically, local or radical nephrectomy remains optimal for local ccRCC or locally advanced ccRCC. In addition to aggressive surgical therapy, cytokine therapy, targeted therapy and novel immunotherapy have positively influenced the survival of patients with this disease 2. However, due to its asymptomatic progression and disappointing laboratory surveillance marker at the early stage, around 20-30% of patients with ccRCC have already been in the invasive and advanced stage at the initial diagnosis 3, and 29% of localized ccRCC patients have nevertheless developed recurrent and metastasis after radical nephrectomy 4. Altogether, such clinical outcomes make it urgent to search for a potential detective and predictive marker for the early diagnosis, risk stratification and selection of treatment modalities.

In this study, we identified 657 differentially expressed proteins by comparing 5 fresh frozen tissues and paired adjacent normal kidney tissues using TMT-LC-MS/MS proteomic technology, and most proteins were enriched in metabolism-related processes. Furthermore, we defined FMNL1 as a potential prognostic marker and confirmed the functional effects of FMNL1 knockdown on the inhibition of proliferation, migration and invasion, which demonstrated essential roles of FMNL1 in the progression of ccRCC.

## Materials and methods

### Clinical specimen preparation

Criteria for the inclusion of patients enrolled in the study:

- Patients with local or locally advanced ccRCC who underwent partial nephrectomy (PN) or radical nephrectomy (RN) at the Department of Urology, Lanzhou University Second Hospital from Jan 2016 to Feb 2019;
- Diagnosed and re-checked with ccRCC by two superior pathologists according to 2012 World Health Organization/International Society of Urological Pathology grading system 5.

Criteria for the exclusion of patients enrolled in the study:

- Patients who received chemotherapy, radiotherapy and immunotherapy before surgery;
- Patients unwilling to sign an informed consent prior to study enrolment;
- Patients with severe neurological and mental disorders and poor compliance;
- Patients with other advanced malignant neoplasm and major cardiovascular diseases.

### Study design

In the discovery set, 5 pairs of fresh ccRCC and adjacent normal tissues were used for proteomic analysis. In the validation set, another 10 pairs of fresh tumor tissues and adjacent normal tissues were used for RT-qPCR/WB assay. IHC assay was performed on 81 ccRCC samples and 16 normal kidney samples. Clinical information of all samples is listed in **Table 1**. The study was approved by the research ethic committee by Lanzhou University Second hospital (No. 2020A-054).

### Protein extraction and TMT-based proteomics

To identify the differentially expressed proteins between ccRCC and adjacent normal tissues, equal amounts of proteins were extracted from cancers (n=5) and corresponding adjacent normal tissues (n=5), respectively. The protein concentration was measured with BCA kit (Beyotime Biotechnology, China) according to the manufacturer's instructions. The tryptic Peptide was reconstituted in 0.5 M TEAB (Sigma, USA) and labelled according to the manufacturer's protocol for TMT kit (Thermo Fisher Scientific, USA). Then labelled peptides were mixed and fractionated into 9 fractions using Agilent 300Extend C18 column (5 μm particles, 4.6 mm ID, 250 mm length), which were dissolved in 0.1% formic acid (solvent A), directly loaded onto a home-made reversed-phase analytical column (15-cm length, 75um i.d.). The peptides were subjected to NSI source followed by tandem mass spectrometry (MS/MS) in Q Exactive TM Plus (Thermo Fisher Scientific, USA) coupled online to the UPLC. The m/z scan range was 350 to 1800 for full scan, and intact peptides were detected in the Orbitrap at a resolution of 70,000. Peptides were then selected for MS/MS using NCE setting as 28 and the fragments were detected in the Orbitrap at a resolution of 17,500.

### Database Search and bioinformatics

The resulting MS/MS data was processed using Maxquant search engine (v.1.5.2.8). Tandem mass spectra were searched against SwissProt Human (20387 sequences) database concatenated with reverse decoy database. FDR was adjusted to < 1% and the minimum score for peptides was set > 40. Protein Annotation, Protein Functional Enrichment, Enrichment-based Clustering and PPI network were executed by online analysis tools.

### Excavation of the TCGA database for survival analysis

The expression values of mRNAs in 525 ccRCC and 72 normal kidney tissues were downloaded from the project TCGA-KIRC on the GDC official website (https://portal.gdc.cancer.gov/). Differential genes were identified on the Networkanalyst Database (https://www.networkanalyst.ca/NetworkAnalyst/home.xhtml) 6. Twenty-nine differentially up-regulated genes illustrated the same change tread with our proteomic results and 2 genes were selected to further study their survival value.

### Validation of FMNL1 expression in human specimens by Real-time qPCR, Western blotting and Immunohistochemical technique

The process of Real-time qPCR was briefly described below. Total RNA from frozen fresh tissues was isolated using Trizol reagent (Thermo Fisher Scientific, Inc.). RNA was reverse transcribed to cDNA using Evo M-MLV RT Kit with gDNA Clean for qPCR (ACCURATE BIOLOGY, Code No. AG11705) on T100^TM^ Thermal Cycler (Bio-Rad Laboratories, Inc.) for 15 minutes at 37 °C, 5 seconds at 85 °C, and stored at 4 °C. FMNL1 and ACTB genes were amplified using CFX96 Real-Time qPCR Detection system (Bio-Rad Laboratories, Inc.) with SYBR-Green PCR Master Mix (Takara Biotechnology Co., Ltd.). The primer sequences were as follow: FMNL1-F 5'-CAGCACCCAAGTCACCGCCAA-3', R 5'-CCCCATCACGGTCGCTCTCA-3'; ACTB-F 5'-CCAACCGCGAGAAGATGACC-3', R 5'-AGCACAGCCTGGATAGCAAC-3'. The relative expression of FMNL1 gene was calculated using 2^-∆∆Ct^ method and normalized to the internal reference gene β-actin.

The brief process of western blotting was shown below. Total proteins were extracted from fresh frozen tissues with lysis buffer. 100mg histocyte lysate were subjected to 10% SDS-PAGE and transferred to PVDF membranes. Blocked with 5% skim milk powder, the membranes were added to primary antibody FMNL1 (ab224247, 1:2500, Abcam, USA; 10395-1-AP, 1:300, Proteintech^TM^, China) and reference proteins shaking 4 °C overnight. Then the membranes were washed by PBS three times and probed with Fluorescein-labeled rabbit derived secondary antibody. After incubation for 1 h at room temperature, Odyssey Near-Infrared Fluorescence Imaging systems (LI-COR Bioscences) and Image Studio Ver 4.0 can be used to observe the results. β-actin was used as a loading control. The loading control sample in each gel was used as a standard for quantification. The optical intensity of each protein band was measured using Image J.

In the IHC assay, to achieve the harmonization standardization during the process of IHC detection, IHC staining was performed using Roche VENTANA BenchMark ULTRA IHC system and sum Integrated Optical Density (IOD) was calculated by Image-Pro Plus 6.0.

### Cell cultures and Lentivirus transduction

In this study, four human cell lines 786-O, Caki-1, HK2 and SNU349 were obtained from Shanghai cell Data. HK2 were cultured in DMEM (L110KJ, L210809, Basal Media), and 786-0, Caki-1, SNU349 were cultured in PRMI 1640 (L210KJ, B210904, Basal Media) with 10% fetal bovine serum (FBS; ABW) and 1% penicillin-streptomycin in a humidified atmosphere containing 5% CO^2^ at 37 °C. The shRNA sequences targeting FMNL1 and negative control sequence were designed and purchased from Shanghai Genechem Co., Ltd. 293T cells were used to generate lentivirus by co-transfecting GV493-shFMNL1s and negative control (CON313, hU6-MCS-CBh-gcGFP-IRES-puromycin) with helper plasmids pHelper 1.0 and pHelper 2.0. The lentiviral knockdown plasmid GV493-shFMNL1s were constructed and used to infect the 786-O and Caki-1 cell lines. The shRNA sequences targeting FMNL1 were listed as follows: shRNA1: ATCCAGACTAAGTTCCGAA, shRNA2: GCGGTTTCAAGTCAAGAAT, negative control sequence: TTCTCCGAACGTGTCACGT. Stable cell lines were selected by puromycin (786-O cells 2.5ug/ml, Caki-1 2ug/ml).

### Cell proliferation, migration, invasion capacities determination

Proliferation ability was evaluated by colony formation assay. Controls as well as lentivirus-infected cells were seeded in 6-well plates at a density of 500 cells and cultured in RPMI 1640 with 10% FBS for 12 days. Formed colonies were fixed with methanol and stained with Gimsa.

Cell migration was calculated by wound healing assay. Controls as well as lentivirus-infected cells were planted in 6-well plates. Straight scratches were produced by a 200 ul pipette tip after cultured cell connectivity reached more than 90%. After incubation in the PRMI 1640 with 10% FBS for 24 h, the wound closure was observed and calculated.

Invasion analysis was performed in Transwell chambers (Corning, USA) coated with Matrigel (BD bioscience, USA) on the upper surface of the 8-um pore size membrane. Two infected cell lines were cultured in Medium with 10% FBS in upper chamber. Medium with 20% FBS was placed in the lower chamber. After incubation for 24 h, the cells that invaded into the lower surface was fixed, stained and counted.

### Statistical analysis

Kaplan-Meier survival curves were plotted to assess the association between FMNL1 expression level and overall survival rate using Log-rank test. Univariate and multivariate Cox proportional hazard model analysis were conducted to estimate the independent prognostic markers. Receiver operating characteristic (ROC) curves were plotted to evaluate the sensitivity and specificity of potential dysregulated genes as diagnostic markers for ccRCC in the TCGA-KIRC datasets. Pearson Chi-Square/Continuity correction test was used to evaluate the relationship between FMNL1 protein level and clinicopathological parameters. Two-way ANOVA was used to evaluate significant differences between the mean values of groups. Statistical significance was set at 2-tailed and P values <0.05. All statistical analysis was performed on SPSS 19.0.

## Results

### Proteomic profiles of ccRCC and adjacent normal kidney tissues

By using multi-whole quantitative proteomic repeated experiments, following the standard of *t*-test p-value<0.05 and fold change higher than 2 or lower than 1/2, 657 differentially expressed proteins were identified, with 186 up-regulated proteins (2.001-8.547 folds) and 471 down-regulated proteins (0.040-0.499 folds) (***Supplementary Table S1****)*.

### Gene Oncology and KEGG pathway enrichment analysis of differentially expressed proteins

**Figure 1:**
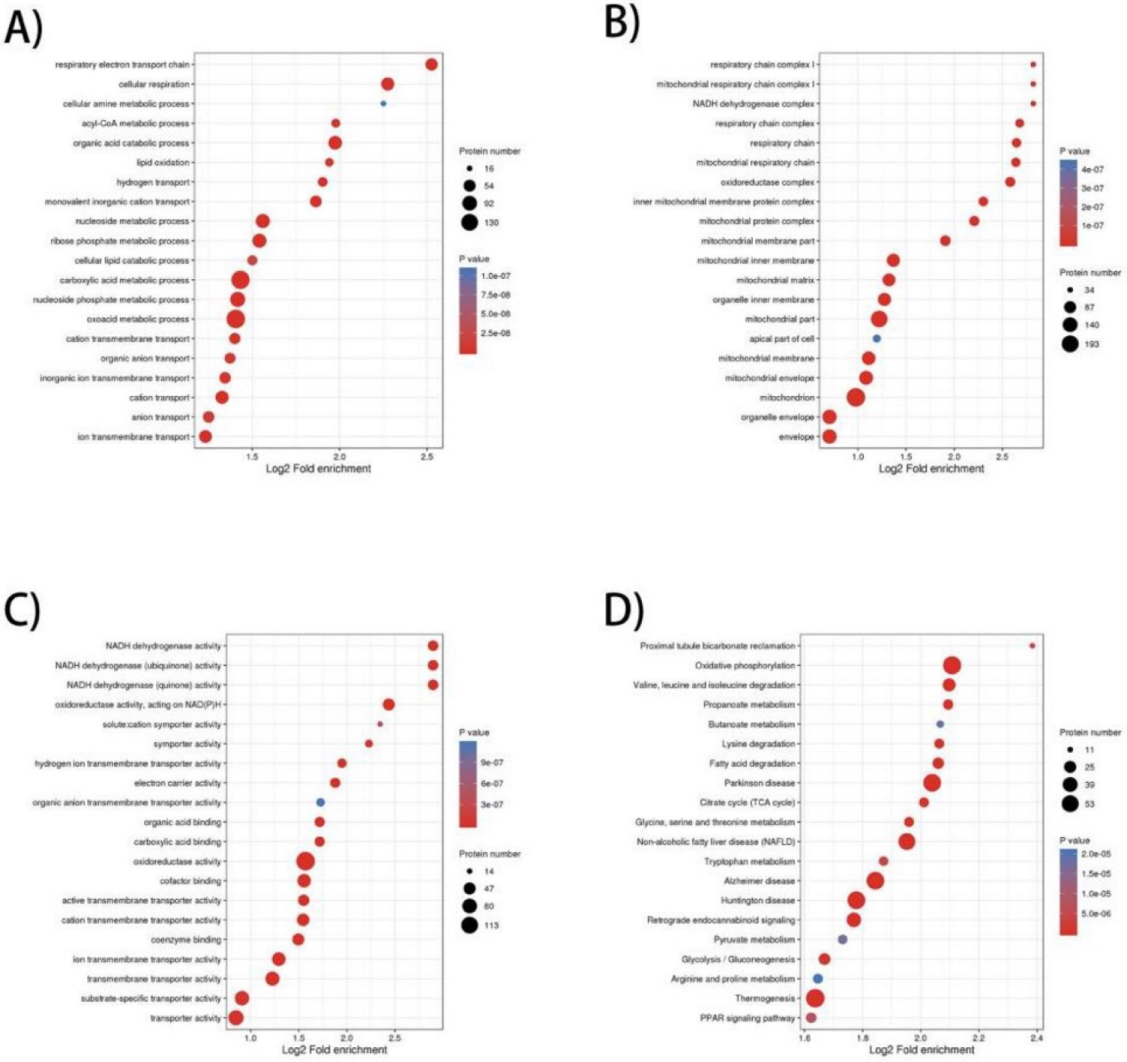
GO (Gene Ontology) and KEGG pathway enrichment bubble plot of differentially expressed proteins in three categories, which shows the top 20significantly enriched GO terms/KEGG pathways. **A)** Biological Process; **B)** Cellular Component; **C)** Molecular Function; **D)** KEGG pathway.

To elucidate the prominent carcinogenesis-related biological processes and pathways, GO enrichment was performed on 657 dysregulated proteins and top 20 significantly enriched processes and pathways was shown in four bubble plots (**Figure 1A-D**). In biological process, the top five most significantly enriched processes were respiratory electron transport chain, cellular respiration, cellular amine metabolic process, acyl-CoA metabolic process and organic acid catabolic process. In molecular function, the top five enrichments were NADH dehydrogenase activity, NADH dehydrogenase (ubiquinone) activity, NADH dehydrogenase (quinone) activity, oxidoreductase activity, acting on NAD(P)H and solute: cation symporter activity. In cellular component, the top five most significantly enriched terms were respiratory chain complex I, mitochondrial respiratory chain complex I, NADH dehydrogenase complex, respiratory chain complex and respiratory chain. GO enrichment analysis of these differentially expressed proteins revealed that dysregulation of mitochondrial function and abnormal transmission of respiratory chain was the main feature of ccRCC compared to normal kidney tissue. In terms of KEGG enrichment analysis, 20 terms were identified as important enriched pathways. Except for the NAFLD, three neurodegenerative diseases and thermogenesis, majority of dysregulated proteins have been implicated in metabolism-related pathways such as oxidative phosphorylation, valine, leucine and isoleucine degeneration, propanoate metabolism, butanoate metabolism, lysine degradation, fatty-acid degeneration, TCA cycle and glycolysis/gluconeogenesis.

### Cluster analysis for the enrichment patterns of GO functional categories and KEGG

**Figure 2:**
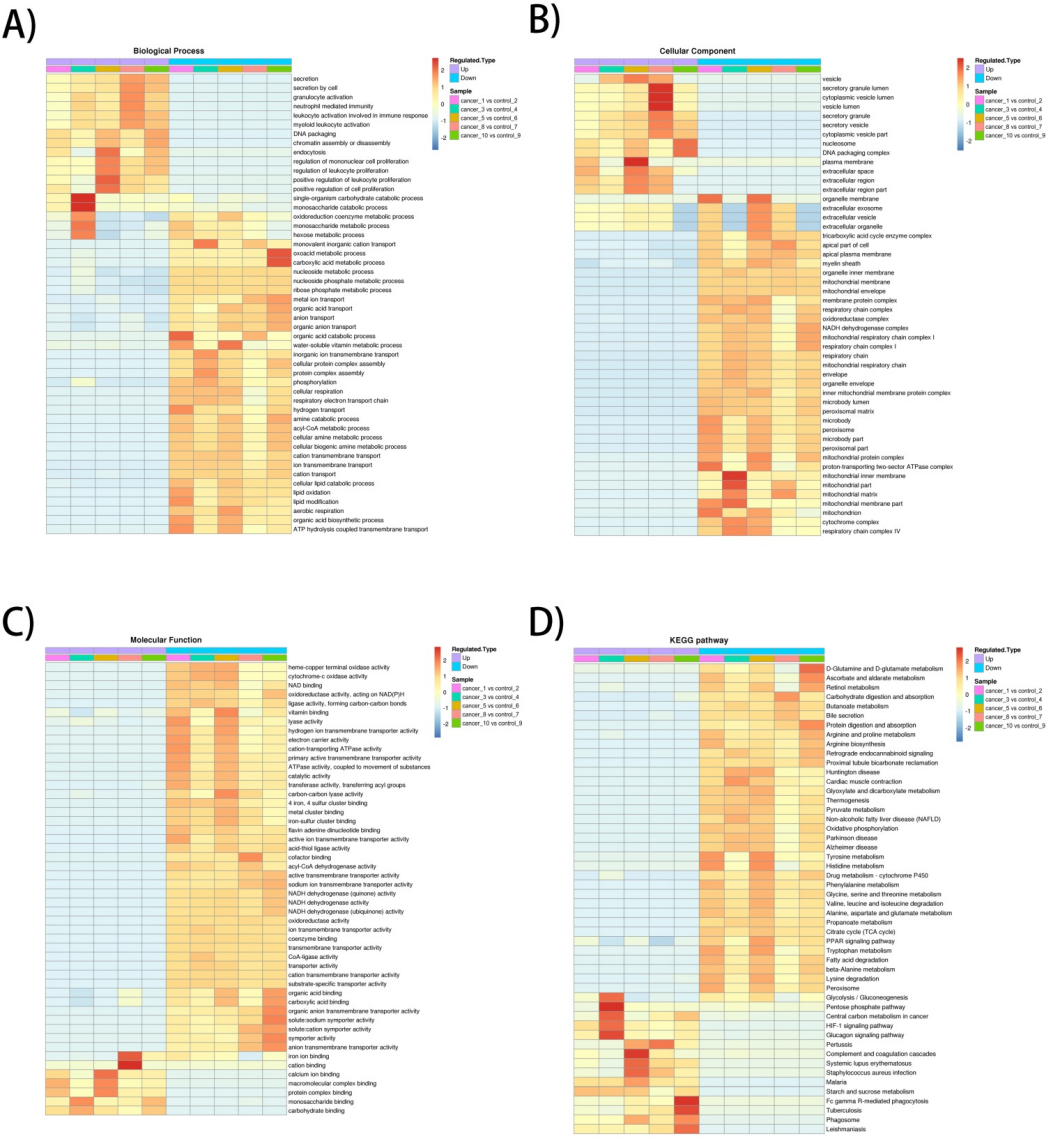
** GO enrichment-based and KEGG pathway-based clustering analysis of the dysregulated proteins. A)** Biological process analysis; **B)** molecular function analysis; **C)** cellular component; **D)** KEGG pathway clustering analysis.

The heatmap of BP enrichment-based cluster analysis showed that the down-regulated proteins were mainly associated with metabolic processes such as aerobic respiration, lipid oxidation, acyl-CoA metabolic process, respiratory electron transport chain, cellular respiration, which most of the processes were associated with mitochondrial oxidative respiration. The up-regulated proteins were mainly involved in proliferation and secretion. In the MF category, most of the down-regulated proteins exhibit enzyme activities related to metabolism and up-regulated proteins were involved in binding activities. The KEGG-based enrichment clustering analysis indicated that glycolysis/gluconeogenesis, pentose phosphate pathway, Central carbon metabolism in cancer and HIF-1 signaling pathway were the major prominent pathways enriched in the up-regulated proteins. Conversely, different amino acids degradation, oxidative phosphorylation, citrate cycle (TCA cycle) and fatty acid degradation and were enriched in down-regulated proteins (**Figure 2A-D**).

### Screening candidate factors with potential prognostic and prognostic value from up-regulated expression

**Figure 3:**
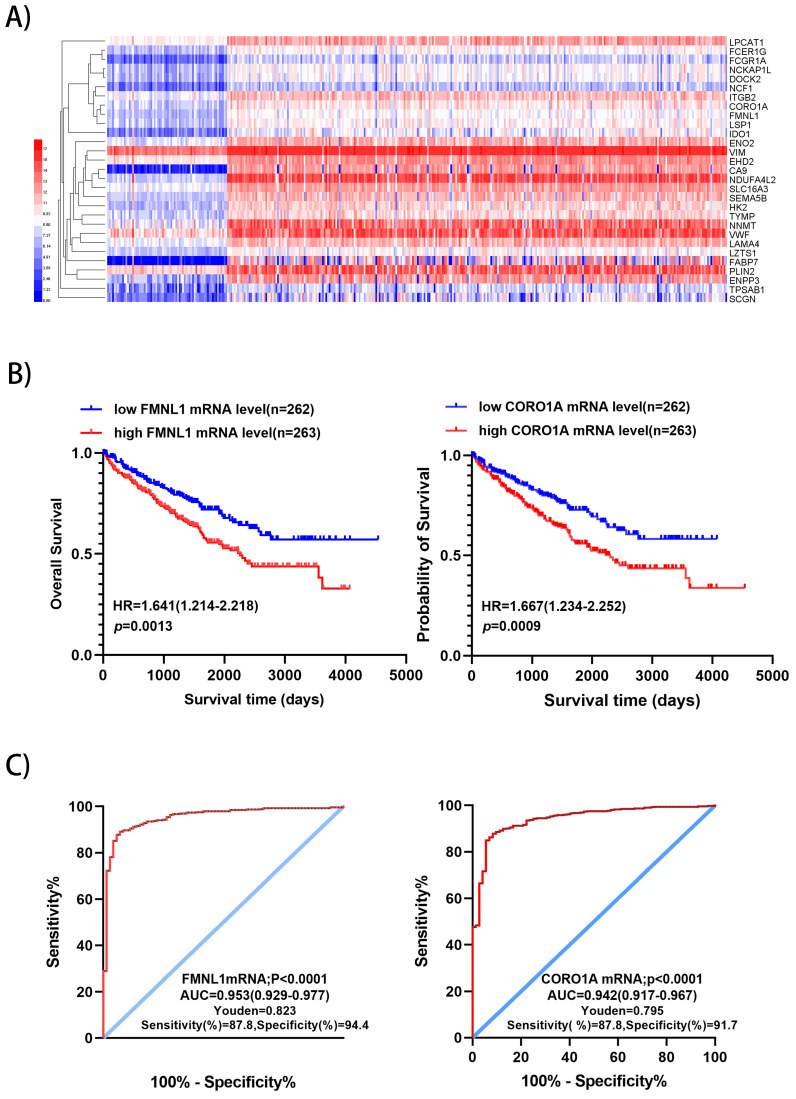
** Validation of 2 potential biomarkers from upregulated expression proteins. A)** Hierarchical clustering analysis of 29 significantly up-regulated genes (FC>5, *P*<0.01) was performed on the Heml 1.0.3.7 derived from TCGA-KIRC datasets. The expression of 29 genes were consistent with our quantitative proteomic results. **B)** Kaplan-Meier curves for OS of patients with ccRCC were performed. Patients with ccRCC were divided into “high”and “low”groups based on median values of genes mRNA sequencing quantification data from TCGA-KIRC project. **C)** ROC curve analysis was performed to evaluate the specificity and sensitivity of the genes FMNL1 and CORO1A for differentiating ccRCC and paired normal tissues in the TCGA-KIRC datasets.

We downloaded the datasets from the project TCGA-KIRC on the GDC (https://portal.gdc.cancer.gov/), which included RNA sequencing data and survival data of 525 ccRCC and 72 normal kidney samples, including 510 significantly up-regulated genes and 752 down-regulated genes were identified with *p*<0.01 and fold change>5 (***Supplementary Table S2***). There were 29 significantly over-expressed genes which were also found in the present proteomic data. Unsupervised hierarchical clustering analysis was performed on the 29 up-regulated genes (**Figure 3A**). Metascape gene annotation tool predicted six genes (FMNL1, NCKAP1L, EHD2, DOCK2, CORO1A, NCF1) participated in actin filament-based process, cortical actin cytoskeleton organization, regulation of cell morphogenesis and cell shape (https://metascape.org). Actin cytoskeleton reassembly and subsequent variation in cell shape provides the structural and morphological basis for cell migration 7. Prognostic value of 6 genes was analyzed by log-rank test and KM survival curves were plotted. Patients with ccRCC were divided into 'high' and 'low' groups based on median values of the genes' mRNA sequencing quantification data from TCGA-KIRC project. The survival curves illustrated that high level of FMNL1mRNA and CORO1A mRNA correlated with worse OS (**Figure 3B**), while high level of of NCKAP1L, DOCK2, EHD2 and NCF1 didn't show any prognostic value (data not shown). Then, ROC curves of FMNL1 and CORO1A in the TCGA-KIRC datasets were plotted, which revealed good sensitivity and specificity for ccRCC prognosis with AUC of 0.953 (95%CI: 0.929-0.977) and 0.942 (95%CI: 0.917-0.967), respectively (**Figure 3C**).

Furthermore, univariate and multivariate analysis were studied to investigate the association of FMNL1 mRNA level, CORO1A mRNA with OS in patients with ccRCC. Univariate analysis indicated that patients with high level of FMNL1mRNA and CORO1A mRNA showed shorter overall survival (FMNL1 mRNA HR: 1.483, *p*<0.000; CORO1A mRNA HR: 1.279, *p*=0.001), while multivariate analysis indicated that only FMNL1 mRNA had negative prognostic value in ccRCC (HR: 1.210, *p*=0.036) (**Table 2**). Taken together, FMNL1 predicted poor prognosis and was a potential independent prognostic marker in patients with ccRCC.

### Gene expression profiles of FMNL1 in human organs and its correlation with cytoskeleton organization

**Figure 4:**
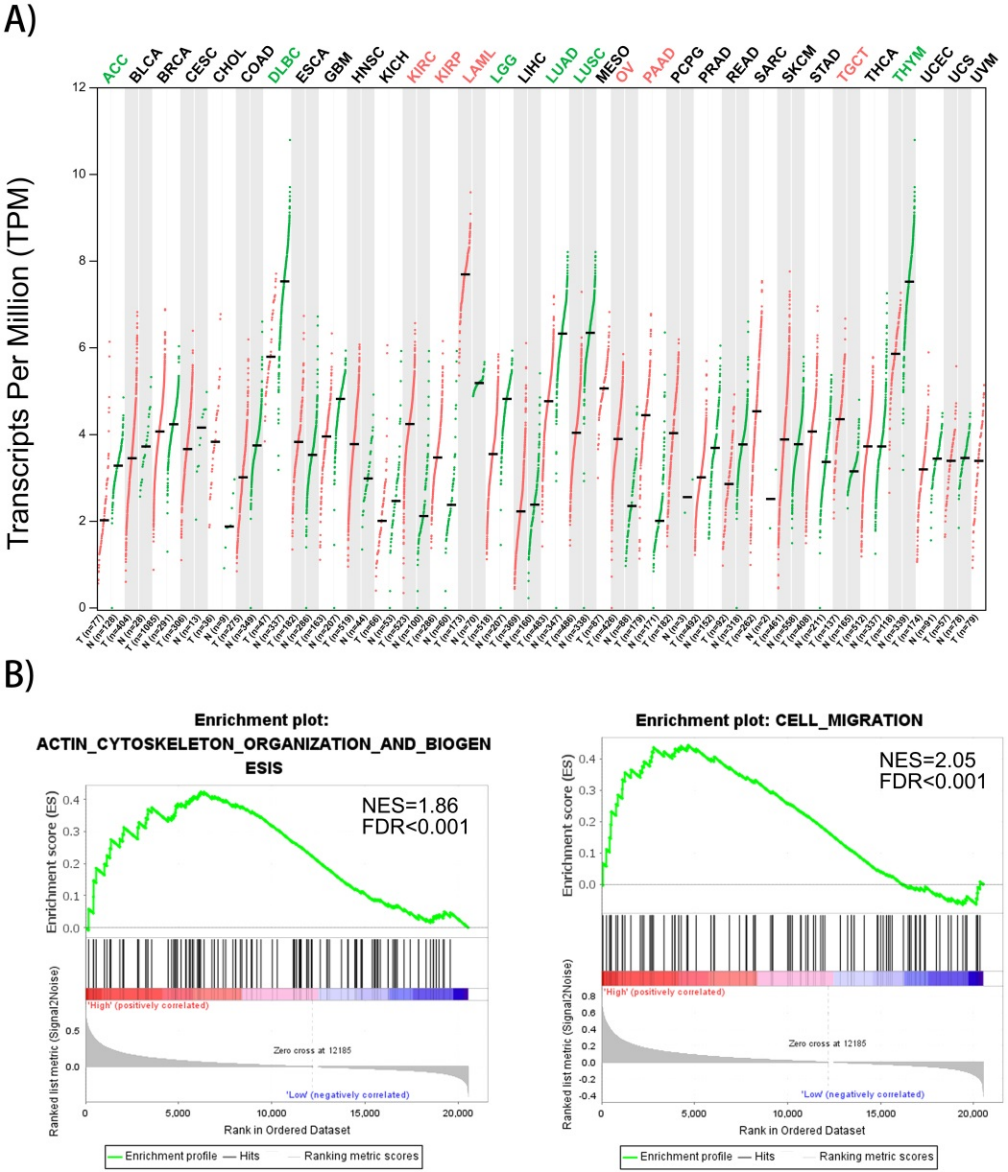
** FMNL1 gene expression profiles and correlated with cell migration. A)** FMNL1 gene expression profiles across all tumor samplesand paired normal tissues based on RNA-Seq data from TCGA database using GEPIA2. **B)** The correlation betwwen FMNL1 mRNA and cell migration and actin cytoskeleton organizaton were analyzed by GSEA assay. |NSE|>1, FDR<0.25,*P*<0.05 were considered as statistically significant.

Gene expression profiles of FMNL1 in human organs was displayed using GEPIA2 8. We found that FMNL1 mRNA in kidney, ovary, pancreas and testis displayed higher expression levels compared with paired normal tissues (**Figure 4A**). To confirm the biological process of FMNL1, GSEA analysis was performed. The results exhibited that the mRNA level of FMNL1 positively correlated with actin-cytoskeleton organization and cell migration (**Figure 4B**).

### Validation of FMNL1 overexpression in an independent ccRCC set and its correlation with clinicopathologic parameters in an expanded validation set

**Figure 5:**
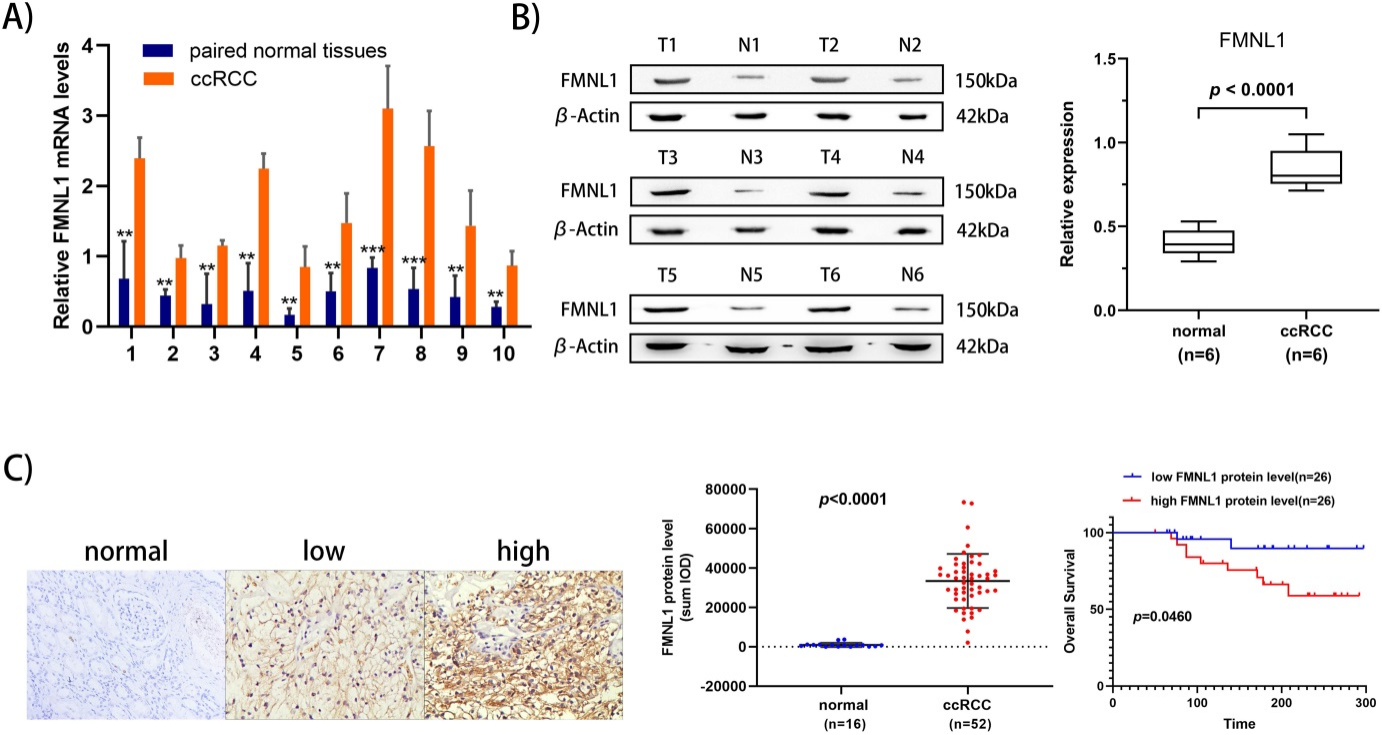
** Validation of FMNL1 expression by RT-qPCR, WB and IHC. A)** Real-time PCR analysis was used to evaluate the expression level of FMNL1 mRNA in 10 paired ccRCC and adjacent normal tissues. Values were presented as the mean±SD.*p<0.05, **p<0.01. **B)** Densitometric analysis and quantification of Western blotting band were performed. The Box plots show the average concentration of FNML1 was higher in ccRCC than in the normal kidney tissues.β-actin was used as a loading control. **C)** IHC was perforemd on 81 tumor tissues and 16 normal tissues (Original magnification x 200).The sum Integrated Optical Density (IOD) of each IHC photo was calculated by Image-Pro Plue 6.0. *P*<0.05 was considered as statistically significant. **D)** Kaplan-Meier analysis presented the correlation between high expression of FMNL1 eand poorer overall survival in 52 ccRCC patients(*p*<0.0001, log-rank test).

To further validate the overexpression of FMNL1 in ccRCC, we examined both the mRNA level and the protein level of FMNL1 in the same cohort of samples. Similar to the results of TCGA_KIRC datasets, an increased FMNL1 mRNA level was observed in 10 tumor samples compared with paired adjacent normal tissues (*p*=0.0012) (**Figure 5A**). Importantly, consistent with our TMT-based proteomic results, WB results further verified the upregulated expression of FMNL1 protein in ccRCCs compared with matched normal kidney tissues (*p*=0.0032) (**Figure 5B**). The expression of FMNL1 protein was also examined by Ventana IHC in 81ccRCC samples and 16 normal kidney tissues. There was positive expression in varying degrees of FMNL1 in 52 ccRCC samples. The expression level of FMNL1 protein was quantified and significantly higher in ccRCC than in normal kidney tissues. Representative microphotograph showed that the positive expression of FMNL1 was localized to the membrane and cytoplasm of cancer cells (**Figure 5C**). Based on median sum optical intensity, 52 positive expressed samples were divided into” high expression” and “low expression” groups. As shown in** Table 3**, there was a strong association between FMNL1 protein level and AJCC stage, Fuhrman grade and metastasis. And KM survival curve illustrated that the patients with high FMNL1 protein level had shorter OS time (*p*=0.0273) (**Figure 5C**). Taken together, these results indicated that FMNL1 protein was significantly associated with advanced stage and shorter OS of ccRCC patients.

### Decreased FMNL1 inhibits the proliferation, migration and invasion capacities of ccRCC cells *in vitro*

**Figure 6:**
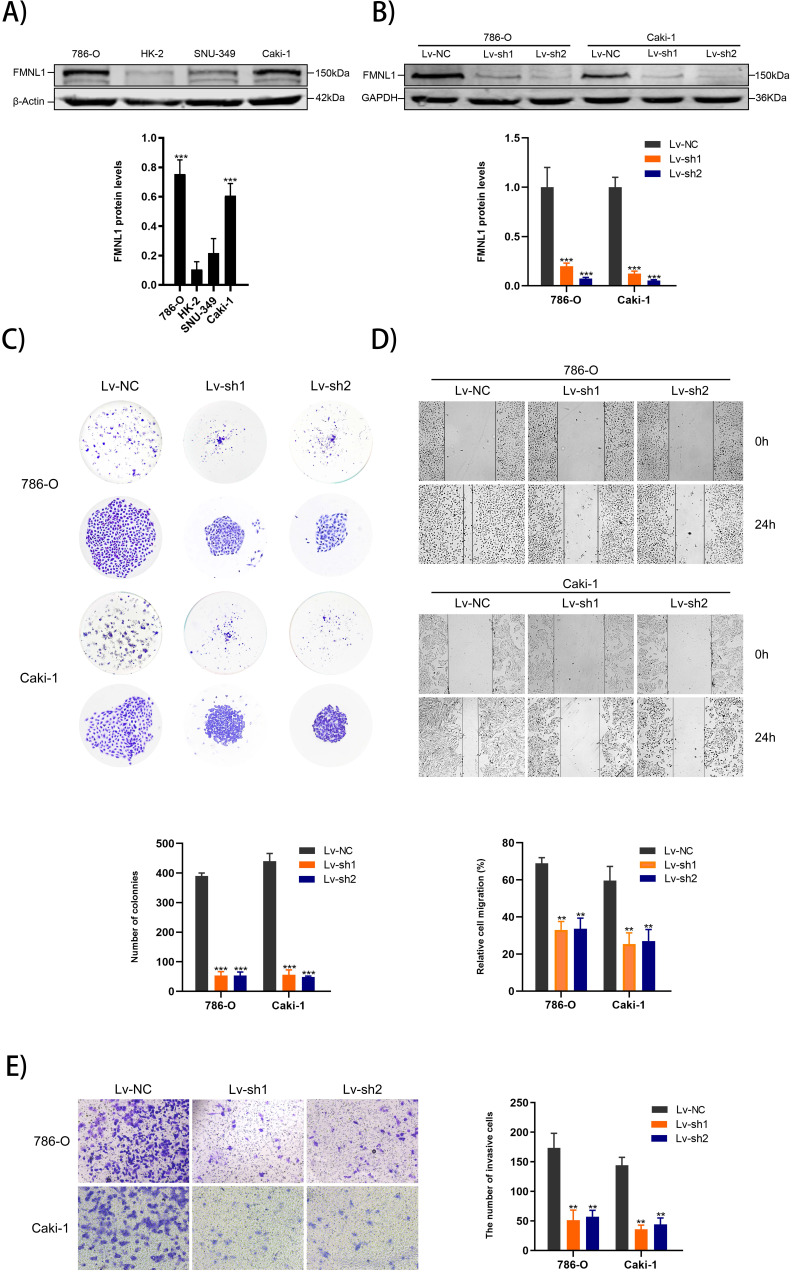
FMNL1 expression in cell lines and FMNL1 knockdown inhibits proliferation, migration and invasion in ccRCC cells. Values were presented as the mean ± SD.*p<0.05, **p<0.01 and ***p<0.001. **A)** Western blot analysis of FMNL1 in normal kidney cell line (HK2) and renal cancer cell lines (786-0, Caki-1 and SNU-349). **B)** Western blot analysis of FMNL1 expression after 786-O and Caki-1 were infected by lentivirus. **C)** Colony formation assay of infected 786-O and Caki-1 cells with FMNL1 knockdown. **D)** Wound healing analysis of infected 786-O and Caki-1 cells with FMNL1 knockdown. **E)** Transwell analysis was performed to calculate the invasive cells in infected 786-O and Caki-1.

Higher expression of FMNL1 protein was observed in 786-0 and Caki-1 than other cell lines, which were thus chosen for the FMNL1 knockdown experiments (**Figure 6A**, *p*<0.005). FMNL1 was downregulated significantly after infected with lentivirus (**Figure 6B**, *p*<0.001). Knockdown of FMNL1 protein decreased the number and size of colony formation in colony formation assays (**Figure 6C**). Would healing analysis and transwell assays indicated that FMNL1 deficiency significantly reduced the migration and invasion ability in ccRCC cell lines (**Figure 6D,E**). Results above demonstrated that reducing FMNL1 expression contributed to suppress the progression of ccRCC.

## Discussion

Along with application of sophisticated mass spectrometry in protein profiling and quantitative analysis, more advanced proteomic technologies (compared with conventional two-dimensional polyacrylamide gel electrophoresis), like TMT (Tandem Mass Tag), SILAC-MS (stable isotope labelling by amino acids in cell culture), iTRAQ (isobaric tags for relative and absolute quantitation) labeling as well as label-free proteomics have been widely used as tools to gain a better understanding of biologic mechanisms responsible for tumor pathogenesis and identify the potential ccRCC-associated biomarkers 9,10,11,12. Various kinds of clinical samples of ccRCC patients can be applied in the proteomic-based technique, including ccRCC cell strain*s,* fresh frozen (FF) tissues, serum, urines, even formalin-fixed paraffin-embedded (FFPE) tissues 10,11,13,14,15. Surgical specimens contain relatively high abundant of dysregulated proteins, and can be collected prospectively in the proteomic analysis or subsequent validation phase. Importantly, randomly mixed samples and biological replicates can eliminate the individual differences to a certain extent. In brief, tissue proteomics, especially fresh frozen tissue proteomics is still a promising alternative strategy to discover and identify ccRCC-related biomarkers.

In our proteomic analysis, 657 dysregulated proteins were identified by TMT-based proteomic approach, including 471 down-regulated proteins and 186 up-regulated proteins. Most quantified proteins were involved in metabolic reprogramming and energy conversion as deciphered in bioinformatic analysis. Gene Ontology enrichment and KEGG pathway analysis demonstrated most up-regulated proteins were enzymes which catalyzed the reactions of glycolysis and central carbon metabolism, while most down-regulated proteins were involved in mitochondrial processes such as oxidative phosphorylation, amino acid metabolism and fatty acid degradation. These results indicated that upregulation of rate-limiting enzymes in glycolysis and mitochondrial impairment were prevalent in cancer cells and may be the cause of metabolic reprogramming in ccRCC.

To evaluate our TMT-MS data, we carried out a comparative study between the present proteomic characterization and 4 previously published comprehensive ccRCC proteomics literatures 11,12,15,16. Of these 4 studies, 2 studies used fresh-frozen specimens for the iTRAQ-based proteomics analysis, and 2 studies used paraffin-embedded tissue or fresh-frozen tissue for the label-free proteomic analysis. In totality, 212, 596, 1055 and 442 differentially expressed proteins were quantified in the four studies, respectively. The comparison showed that 156 (156), 207 (206), 356 (352) and 253 (253) differentially expressed proteins quantified in our proteomic data were detected in the four previous research respectively (numbers in brackets represent the proteins with the same expression trend as our proteomic data). A total of 426 DEPs in our data were detected in 4 previous studies, which accounted for 64.8% of the 657 dysregulated proteins. GO and Pathway analysis of differentially abundant proteins in each study was performed to provide biological annotation. More importantly, all 4 previous studies confirmed that dysregulated metabolic pathways were the top significantly enriched processes. Briefly, in agreement with our proteomic results, the studies mentioned above revealed that reprogramming energy metabolism was one of the most distinct characteristics in ccRCC.

Through the strategy of searching for the common DEPs in proteomic data and transcriptomic profiling from TCGA, we identified FMNL1 as a potential prognostic marker by exploring dysregulated proteins annotated with the actin-cytoskeleton organization and cell migration. GO enrichment and GSEA assay confirmed the association of FMNL1 with actin cytoskeleton organization and cell migration. The migration of tumor cells from primary tumors, entry to blood or lymphatic vessels and growth in distant tissues or organs are understood as typical process of tumor invasion and metastasis 17. FMNL1 belongs to a member of formin family of proteins and have been implicated in the regulation of cell morphology, cytoskeletal organization and cell migration 18. As reported before, FMNL1 was upregulated in various malignancies, like lymphoma, T non-Hodgkin's lymphomas, GBM (Glioblastoma multiforme), NSCLC and NPC 19,20,21*.* Involvement of FMNL1 in the physiological mechanism of malignancies has been reported. The variant expression of FMNL1 was correlated with invasion and migration of the GBM cell *in vitro* experiment 19. A series of *in vitro* and* in vivo* assays illustrated that inhibition of FMNL1 expression could reduce the proliferation, invasion and migration of NSCLC cell lines, and restrained the metastasis ability of cell lines 20. In the study of nasopharyngeal carcinoma, *in vivo* and* in vitro* results also illustrated that overexpression of FMNL1 promoted cell migration, invasion, invadopodia formation, and enhanced aggressiveness ability of NPC cells 21. Correlation also existed between the overexpression of FMNL1 and poor patient survival in GBM, NSCLC and NPC.

In our validation tests, we proved FMNL1 was upregulated in ccRCC tissues compared with adjacent normal kidney tissues. The over-expression of FMNL1 was significantly correlated with tumor stage and distant metastasis. All these results revealed that high expression level of FMNL1 was associated with the advanced stage of ccRCC and poor clinical outcome of ccRCC patients. A series of *in vitro* experiments revealed that depletion of FMNL1 in 786-O and Caki-1 impaired the proliferation, migration and invasion capacities, respectively, which demonstrated that inhibiting FMNL1 expression contributed to suppress the progression of ccRCC significantly and might be a new effective therapeutic strategy. Nevertheless, how reduced FMNL1 expression hindered the progression of ccRCC, further study is still required.

## Conclusion

In conclusion, we used TMT-labeling proteomic approach to provide a comprehensive protein signature of ccRCC and revealed its sophisticated metabolic reprogramming. Moreover, FMNL1 has been identified as a prognostic marker, and functional effect of FMNL1 on proliferation, migration and invasion capacities of ccRCC cell lines illustrated that FMNL1 might be a new effective therapeutic strategy to inhibit the progression of ccRCC.

## Supplementary Material

Supplementary table 1.

Supplementary table 2.

## Figures and Tables

**Table 1:** Clinicopathological characteristics of samples with ccRCC in different subjects

Clinico-pathological features (No. of samples)	Discovery set^a^ by TMT	Validation set from TCGA_KIRC datasets	Further validation set by WB^b^/RT-PCR	Expanded validation set by Ventana IHC
	5 pairs of ccRCC and adjacent normal tissues	525 ccRCC and 72 adjacent normal tissues	10 pairs of ccRCC and adjacent normal tissues	81 ccRCC and 16 normal tissues
**Gender**				
Male	2 (40.0%)	343 (65.3%)	4 (40.0%)	47 (58.0%)
Female	3 (60.0%)	182 (34.7%)	6 (60.0%)	34 (42.0%)
**Age at surgery**				
<60	2 (40.0%)	245 (46.7%)	3 (30.0%)	39 (48.1%)
≥60	3 (60.0%)	280 (53.3%)	7 (70.0%)	42 (51.9%)
**Histologic grade** ^c^				
low (1-2)	3 (60.0%)	not reported	8 (80.0%)	43 (53.1%)
high (3-4)	2 (40.0%)	not reported	2 (20.0%)	38 (46.9%)
**Tumor extent** ^d^				
Tx/T1 or T2	3 (60.0%)	336 (64.0%)	9 (90.0%)	65 (80.2%)
T3 or T4	2 (40.0%)	189 (36.0%)	1 (10.0%)	16 (19.8%)
**Lymph node metastasis** ^d^				
Nx or N0	4 (80.0%)	509 (97.0%)	10 (100%)	67 (82.7%)
N1	1 (20.0%)	16 (3%)	0	14 (17.3%)
**Distant metastasis** ^d^				
M0	5 (100%)	447 (85.1%)	9 (90.0%)	65 (80.2%)
M1	0	78 (14.9%)	1 (10.0%)	16 (19.8%)
**AJCC stage** ^d^				
I or II	3 (60%)	318 (60.6%)	7 (70.0%)	64 (79.0%)
III or IV	2 (40%)	207 (39.4%)	3 (30.0%)	17 (21.0%)
**Recurrence**	-	-	-	10 (12.3%)
**Median Follow-up** (range)	-	1200 days (3-4537)	-	179.5 weeks (64-292)
**No. of death**	-	170 (32.4%)	-	11 (13.6%)

^a^ Proteomic study was performed using 5 paired frozen ccRCC and adjacent normal samples. ^b^Western blotting assay was performed on 6 out of 10 paired frozen ccRCC and adjacent normal kidney tissues. ^c^Fuhrman Pathological nuclear grade. ^d^Tumor staging was based on the 8th Edition of the AJCC TNM staging of renal tumors.

**Table 2:** Univariate and multivariate analysis of FMNL1 mRNA level and patient survival

Clinico-pathological parameters	Univariate analysis			Multivariate analysis		
	HR^a^	*95%Cl^b^*	*P*	HR	*95%Cl*	P^*^
**Age**						
>60 (n=280)	1.030	1.017-1.044	0.000	1.034	1.020-1.049	**0.000**
≤60 (n=245)						
**Gender**						
Male (n=343)	1.039	0.759-1.421	0.813	-	-	-
Female (n=182)						
**Tumor extent**						
Tx/1/2 (n=336)	3.189	2.345-4.336	0.000	0.894	0.491-1.627	0.714
T3/4 (n=189)						
**Lymph node metastasis**						
Nx or N0 (n=509)	3.893	2.107-7.193	0.000	1.870	0.955-3.660	0.068
N1 (n=16)						
**Distant metastasis**						
M0 (n=447)	4.442	3.251-6.070	0.000	2.560	1.763-3.718	**0.000**
M1 (n=78)						
**AJCC Stage**						
I or II (n=318)	3.938	2.855-5.433	0.000	2.487	1.247-4.960	**0.010**
III or IV (n=207)						
**FMNL1 mRNA**						
Low (n=262)	1.483	1.243-1.769	0.000	1.210	1.013-1.445	**0.036**
High (n=263)						
**CORO1A**						
Low (n=262)	1.279	1.105-1.481	0.001	1.064	0.916-1.235	0.419
High (n=263)						

^a^Hazard ratio was calculated from Cox proportional hazard regression model. ^b^Confidence Interval of HR. ^*^p<0.05 was considered as significant.

**Table 3:** Correlation between FMNL1 proten expression level and clinicopathological parameters of ccRCC

Clinicopathological Parameters	n	FMNL1 protein expression level		χ^2^	p^*^
		Low	High		
**Age**					
<61	29	18	11	3.820	0.051
≥61	23	8	15		
**Gender**					
Male	28	16	12	1.248	0.266
Female	24	10	14		
**T stage**					
T1, T2	38	20	18	2.383	0.123
T3, T4	14	4	10		
**N stage**					
N0	40	22	18	1.733	0.188
N1	12	4	8		
**M stage**					
M0	42	24	18	4.457	**0.035**
M1	10	2	8		
**AJCC stage**					
I or II	36	22	14	5.778	**0.016**
III or IV	16	4	12		
**Fuhrman grade**					
1,2	25	17	8	6.240	**0.012**
3.4	27	9	18		

*p<0.05 was considered as significant.

## Abbreviations

ccRCC: clear cell renal cell carcinoma
FMNL1: Formin-like protein 1
CORO1A: Coronin 1A
TMT: Tandem Mass Tag
LC-MS/MS: Liquid chromatography-mass spectrometry/mass spectrometry
TCGA: The Cancer Genome Atlas
IHC: immunohistochemical technique
EDTA: Ethylene Diamine Tetraacetic Acid
TEAB: Triethylammonium Bicarbonate
TFA: Trifluoroacetic Acid
GSEA: gene set enrichment analysis
GEPIA2: Gene Expression Profiling Interactive Analysis
ROC: Receiver operating characteristic
AUC: Area of Under Curve
FFPE: formalin-fixed paraffin-embedded
